# Gelatin-Based Hydrogels for the Controlled Release of 5,6-Dihydroxyindole-2-Carboxylic Acid, a Melanin-Related Metabolite with Potent Antioxidant Activity

**DOI:** 10.3390/antiox9030245

**Published:** 2020-03-18

**Authors:** Maria Laura Alfieri, Giovanni Pilotta, Lucia Panzella, Laura Cipolla, Alessandra Napolitano

**Affiliations:** 1 Department of Chemical Sciences, University of Naples Federico II, I-80126 Naples, Italy; marialaura.alfieri@unina.it (M.L.A.); panzella@unina.it (L.P.); 2 Department of Biotechnology and Biosciences, University of Milano-Bicocca, I-20126 Milan, Italy; g.pilotta@campus.unimib.it

**Keywords:** 5,6-dihydroxyindole-2-carboxylic acid, gelatin, cross-linked hydrogel, antioxidant activity, controlled release

## Abstract

The ability of gelatin-based hydrogels of incorporating and releasing under controlled conditions 5,6-dihydroxyindole-2-carboxylic acid (DHICA), a melanin-related metabolite endowed with marked antioxidant properties was investigated. The methyl ester of DHICA, MeDHICA, was also tested in view of its higher stability, and different solubility profile. Three types of gelatin-based hydrogels were prepared: pristine porcine skin type A gelatin (HGel-A), a pristine gelatin cross-linked by amide coupling of lysines and glutamic/aspartic acids (HGel-B), and a gelatin/chitosan blend (HGel-C). HGel-B and HGel-C differed in the swelling behavior, showed satisfactorily high mechanical strength at physiological temperatures and well-defined morphology. The extent of incorporation into all the gelatins tested using a 10% *w/w* indole to gelatin ratio was very satisfactory ranging from 60 to 90% for either indoles. The kinetics of indole release under conditions of physiological relevance was evaluated up to 72 h. The highest values were obtained with HGel-B and HGel-C for MeDHICA (90% after 6 h), and an appreciable release was observed for DHICA reaching 30% and 40% at 6 h for HGel-B and HGel-C, respectively. At 72 h, DHICA and MeDHICA were released at around 30% from HGel-A at pH 7.4, with an increase up to 40% at pH 5.5 in the case of DHICA. DHICA incorporated into HGel-B proved fairly stable over 6 h whereas the free compound at the same concentration was almost completely oxidized. The antioxidant power of the indole loaded gelatins was monitored by chemical assays and proved unaltered even after prolonged storage in air, suggesting that the materials could be prepared in advance with respect to their use without alteration of their efficacy.

## 1. Introduction

5,6-Dihydroxyindole-2-carboxylic acid (DHICA) is a key intermediate in the tyrosinase catalyzed oxidation of tyrosine leading to eumelanins, the main epidermal human pigments responsible for skin photoprotection. The levels of DHICA are dictated by the activity of the enzyme dopachrome tautomerase or tyrosinase related protein-2, that favors the non-decarboxylative rearrangement pathway of dopachrome, determining a significant incorporation of this indole in natural eumelanins and its presence at nM levels in blood and body fluids in the form of two methylated metabolites, 6-hydroxy-5-methoxyindole-2-carboxylic acid and 5-hydroxy-6-methoxyindole-2-carboxylic acid [1]. In addition to DHICA, other melanin precursors like tyrosine and 3,4-dihydroxyphenylalanine (DOPA) have been shown to exert important role acting as inducers and regulators of the melanogenic apparatus and of MSH receptors [2,3].

In the last decade, several evidences have accumulated indicating that DHICA may exert an antioxidant and protective function *per se* unrelated to pigment synthesis. Early studies showed that DHICA inhibits lipid peroxidation in vitro [4]. Subsequent works indicated that DHICA is oxidized by nitric oxide and efficiently inhibits H_2_O_2_-Fe(II)/EDTA (Fenton)-induced oxidation processes [5,6]. Moreover, DHICA exhibits excellent triplet quenching properties [7]. DHICA has also an intense absorption maximum at 313 nm, in the erythemigenic UVB region, and exhibits efficient excited state relaxation mechanisms of potential relevance to UV dissipation [8]. 

The antioxidant profile characterized in different in vitro assays suggested that it may act as a diffusible protective mediator under oxidative stress conditions [9]. In addition, studies on primary cultures of human keratinocytes disclosed its remarkable protective and differentiating effects [10]. At micromolar concentrations, DHICA induced: (a) time- and dose-dependent reduction of cell proliferation without concomitant toxicity; (b) enhanced expression of early and late differentiation markers; (c) increased activities and expression of antioxidant enzymes; and (d) decreased cell damage and apoptosis following UVA exposure. Similarly to DHICA, other structurally related compounds were reported to ensure effective protection of cutaneous homeostasis from hostile environmental factors [11].

All together these results suggest a high potential of DHICA for applicative purposes including treatment of disorders and pathological conditions affecting skin and mucous membranes. Severe limitations in this perspective stem from the ease of this compound to undergo oxidation with subsequent loss of its properties. In addition, proper formulation allowing for vehiculation through the skin and a controlled release would greatly add to the beneficial effects prolonging the action and taking the bioavailable concentrations relatively low. 

In recent years, several natural compounds have been tested for the topical treatment of skin disorders by use of a variety of transcutaneous delivery systems including lipophilic nanoparticles like liposomes [12], solid lipid nanoparticles [13,14], nanostructured lipid carriers, monoolein aqueous dispersions [15,16], ethosomes [17,18], and lecithin organogels [19,20]. Speed up of wound healing process by a nanohydrogel embedding an antioxidant compound like baicalin has been described that exhibited optimal performance for a complete skin restoration and inhibition of specific inflammatory markers [21].

A variety of hydrophilic delivery systems have also been explored such as gelatin, the product of collagen hydrolysis, as it offers several advantages including the historical safe use in a wide range of medical applications, low costs, inherent electrostatic binding properties, and proteolytic degradability. In addition, gelatin versatility allows the design of different carrier systems, spanning from micro or nanoparticles, to fibers and hydrogels. Hydrogel based scaffolds are largely applied in the field of tissue engineering since their mechanical features can be tuned to the tissue being repaired and because they offer 3D networks able to support cell growth, differentiation, and migration.

Several reports have described the ability of gelatin hydrogels to adsorb bioactive molecules and/or drugs into the polymer network, thus allowing their controlled release, e.g., for pain treatment and wound healing and tissue regeneration applications [22,23].

In order to design gelatin-based systems, to proper tuning the mechanical properties, swelling behavior, thermal properties, and other physiochemical properties [24,25], gelatin is usually cross-linked by chemical, enzymatic or physical methods and many different approaches have been proposed in the last years. As for the chemical cross-linking, one may rely directly on the chemistry of amino acid side chains by coupling reactions (i.e., amide bond formation) or cross-linking by homo- or heterofunctional cross-linkers (i.e., glutaraldehyde [26], genipin [27], triazolinedione [28]) or convert them into suitable functionalities in order to exploit different chemistries (i.e., photopolymerizable acrylates [29,30] and thiol-ene chemistry [31]).

In addition, gelatin-based hydrogels may be further improved by the use of blends in combination with natural or synthetic polymers such as polyglutammic acid [32,33], chitosan [34], or polyvinyl alcohol [35].

Within this framework in the present work, we have investigated the ability of gelatin-based hydrogels of incorporating and releasing under controlled conditions DHICA. The methyl ester of DHICA, MeDHICA, was also tested in view of its higher stability and different solubility profile [36]. Three different type of gelatin-based hydrogels were prepared as drug release systems: (a) pristine porcine skin type A gelatin (HGel-A); (b) pristine gelatin cross-linked by amide coupling of lysines and glutamic/aspartic acids (HGel-B), and (c) a gelatin/chitosan blend (HGel-C). 

These gelatin-based hydrogels could have different potential applications e.g., for topical uses or as scaffolds for cellular growth. In all cases, a satisfactory loading and a smooth release at physiological pH of DHICA and its methyl ester were observed while chemical assays confirmed the antioxidant power of the indole loaded gelatin hydrogels. 

## 2. Materials and Methods 

Gelatin type A from porcine skin (gel strength ~300 g bloom), 4-(4,6-dimethoxy-1,3,5-triazin-2-yl)-4-methylmorpholinium-chloride (DMTMM), chitosan, glacial acetic acid, 2,2-diphenyl-1-picrilhydrazyl (DPPH), ferric chloride (III) hexahydrate, and 2,4,6-tris(2-pyridyl)-*s*-triazine (TPTZ) were purchased from Sigma Aldrich (Milano, Italy) and used without any further purification. Phosphate buffer saline (PBS) 10× was purchased from VWR. DHICA and MeDHICA were prepared according to a procedure previously developed [36,37].

The UV–Vis spectra were recorded on a Jasco V-730 Spectrophotometer (Lecco, Italy).

HPLC analyses were performed on an Agilent 1100 binary pump instrument (Agilent Technologies, Milan, Italy) equipped with a SPD-10AV VP UV–visible detector using an octadecylsilane-coated column, 250 mm × 4.6 mm, 5 μm particle size (Phenomenex Sphereclone ODS, Bologna, Italy) at 0.7 mL/min. Detection wavelength was set at 300 nm. Eluant system: 1% formic acid:acetonitrile, 85:15 *v*/*v*.

### 2.1. Hydrogels Preparation

#### 2.1.1. Pristine Gelatin Hydrogel (HGel-A)

A total of 0.2 g of gelatin was dissolved and mixed in 2 mL (10% *w*/*v*) of PBS (pH = 7.4) at 37 °C. After 5 min, DHICA or MeDHICA pre-dissolved in the minimal amount of DMSO were added to the gelatin solution up to 1, 5, 10% *w*/*w* with respect to gelatin in the case of DHICA, or 10% *w*/*w* in the case of MeDHICA, and continuously stirred until the solution appeared homogeneous. The hydrogels were set for gelation for 12 h at 4 °C and then washed with 5 mL PBS (pH = 7.4) for 30 min to remove not incorporated indole compounds.

#### 2.1.2. Cross-Linked Pristine Gelatin Hydrogel (HGel-B)

For preparation of HGel-B (10% *w*/*v*), gelatin (1 g) was initially dispersed in 10 mL of PBS, pH = 7.4, at 45 °C with continuous stirring till complete dissolution (1.5 h). DMTMM was then added (44 mg, 0.16 mmol dissolved in 100 µL of PBS, roughly 20% mmol of gelatin carboxyl groups), and the solution was kept under stirring at 45 °C for 30/45 s. Finally, the solution was poured in a 24 multiwell plate (1 mL per well), plugged, and rested till gelation (5–10 min); the gels were then kept at 37 °C for 2 h and finally freeze-dried.

#### 2.1.3. Gelatin-Chitosan Blend (HGel-C) 

For preparation of HGel-C (gelatin/chitosan 8:1 *w*/*w*), 0.8 g of gelatin was initially dispersed in 8 mL (10% *w*/*v* gelatin solution) of PBS, pH = 7.4, and maintained at 45 °C under stirring till complete dissolution (1.5 h). Chitosan (0.1 g) was dissolved in 0.833 mL of 0.5 M acetic acid till complete dissolution (3 h). Gelatin solution was poured into the chitosan solution and kept under stirring at 45 °C. After 24 h, DMTMM (18 mg, 0.064 mmol dissolved in 70 µL of PBS, roughly 10% mmol based on the gelatin carboxyl groups) was added. The solution was poured in cylindric molds (17 mm in diameter with total volume of 2.2 mL, 1.7 mL/mold), plugged, and rested till gelation (5–10 min); the gels were then kept at 37 °C for 2 h. Finally, the gels were dried at 4 °C.

### 2.2. Determination of Swelling Degree of HGel-B and HGel-C

Washed hydrogels were swollen in distilled water up to 5 h and the swollen weight was recorded at 10 min intervals, after dabbing the hydrogels with a filter paper before weighing. Totally, three replicas were run. The degree of swelling (*SD_i_*) was calculated as the following:

*SD_i_* = [(*Mw_i_* – *Md*)/*Md*] × 100% (1)

where *Mw_i_* is the swollen weight and *Md* is the dry weight [38].

### 2.3. Fourier Transform-Infrared Spectroscopy (FT-IR)

FT-IR analyses of loaded and unloaded gelatins HGel-B and HGel-C were done in the Attenuated Total Reflectance (ATR) mode using a Thermo Fisher Nicolet 5700 spectrophotometer equipped with a Smart Performer accessory (Rodano, Italy) mounting a ZnSe crystal for the analysis of solid samples.

### 2.4. Scanning Electron Microscopy (SEM)

The surface morphology of gelatins HGel-B and HGel-C were examined using a SEM Tabletop Microscope-1000 and a Field Emission SEM (FEI corporate, Hillsboro, OR, USA). The solid samples were mounted on a stub using double-sided adhesive tape before being coated with gold.

### 2.5. Loading of DHICA/MeDHICA to HGel-B and HGel-C Gelatins

HGel-B and HGel-C were swelled in distilled water for 1 h or 4 h, respectively. After that DHICA or MeDHICA, pre-dissolved in the minimal amount of DMSO, were added to the gelatin solution in PBS 1× (pH = 7.4) up to 10% *w*/*w* with respect to gelatin. In the case of HGel-B, DHICA was also used at 5% *w*/*w* with respect to gelatin. The optimum loading time was determined by UV–Vis monitoring of the remaining indole in the solution.

### 2.6. Kinetics of DHICA/MeDHICA Release

#### 2.6.1. HGel-A

The kinetics of release of the indoles in PBS 1× at pH 7.4 or 5.5 was evaluated at room temperature by UV–Vis analysis over 72 h by refreshing the medium every 1 h in the first 6 h and then every 24 h.

#### 2.6.2. HGel-B and HGel-C

The kinetics of release of indole componds (DHICA 5, 10% or MeDHICA 10% from HGel-B and DHICA/MeDHICA 10% from HGel-C) were determined in PBS 1× (pH = 7.4) at 37 °C by UV–Vis analysis over time. The medium was repeatedly refreshed every hour over 6 h. 

### 2.7. Stability of DHICA/MeDHICA

Then, 10% HGel-B incorporating DHICA was immersed in PBS 1× at 37 °C and the release of the indole was monitored over 6 h without medium refreshing. The decay of free DHICA in the PBS solution at 37 °C at the same concentration (based on the estimated incorporation of DHICA in the gelatin as described above) was monitored by HPLC analysis.

### 2.8. Antioxidant Properties of the DHICA/MeDHICA Loaded Gelatins

#### 2.8.1. DPPH Assay

The assay was performed as previously described [39]. Briefly, HGel-B and HGel-C, loaded with DHICA or MeDHICA at 10% *w*/*w*, were immersed at 37 °C (10 mg/mL) in PBS medium. Then, 60 µL aliquots were periodically withdrawn over 6 h and added to 200 μM DPPH solution in methanol (2 mL) with rapid mixing. The reaction was followed by spectrophotometric analysis measuring the absorbance at 515 nm after 10 min. Values are expressed as DPPH decay over time. The experiments were run in triplicates. In other experiments DHICA or MeDHICA loaded gelatins that had been taken in air over one week were immersed in 200 μM DPPH solution (using a 0.04 *w*/*v* ratio) and the antioxidant power was evaluated by UV–Vis recording spectra over time up to 7 days. In control experiments the DPPH assay was run on the materials soon after loading of the indoles.

#### 2.8.2. Ferric Reducing/Antioxidant Power (FRAP) Assay

The assay was performed as previously described [40]. Briefly, the ferric reducing/antioxidant power (FRAP) reagent was prepared by sequentially mixing 0.3 M acetate buffer (pH = 3.6), 10 mM TPTZ in 40 mM HCl, and 20 mM ferric chloride in water, at a 10:1:1 *v*/*v*/*v* ratio. To a solution of the FRAP reagent, 12 μL aliquots of the PBS medium, in which 10% HGel-B and HGel-C had been immersed at 37 °C, were added and rapidly mixed. After 10 min, the absorbance at 593 nm was measured. The assay was repeated on the samples over 6 h.

### 2.9. Statistical Analysis

In all the experiments, each sample was tested in three independent analyses, each carried out in triplicate. The results are presented as the mean ± SD values obtained.

## 3. Results and Discussion

### 3.1. Loading of Indole Compounds in Gelatin and Release Kinetics

**Figure 1:**
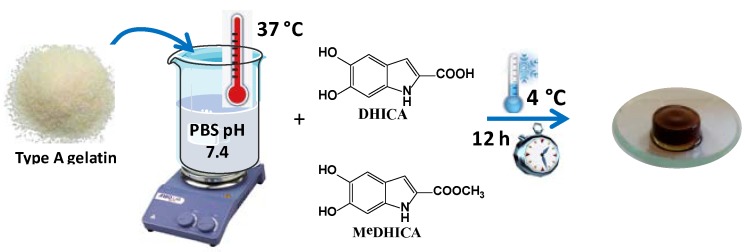
Preparation of gelatin and loading of indole compounds.

In the initial experiments, porcine skin gelatin type A was dissolved at 10% *w*/*v* concentration in PBS at pH 7.4 at 37 °C (HGel-A) and DHICA or MeDHICA previously dissolved in the minimal amount of DMSO, were added under stirring to a 10% *w*/*w* concentration with respect to gelatin (10% *w*/*w* DHICA or MeDHICA/HGel-A). The solutions were set for gelation for 12 h at 4 °C and then washed with PBS to remove not incorporated indole compounds (HGel-A, Figure 1) [32].

UV–Vis spectrophotometric analysis of the indoles (λ_max_ 320 nm) in the washings allowed to estimate an extent of incorporation into the gelatins of 62 ± 1.7% in the case of DHICA and even higher, up to 80 ± 1.3%, for MeDHICA. Using DHICA at 1 or 5% *w/w* concentration with respect to gelatin, the extent of incorporation proved to be 48 and 59%, respectively. 

**Figure 2:**
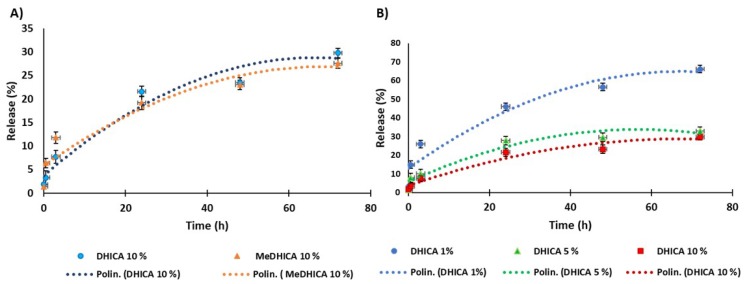
Release kinetics of 5,6-dihydroxyindole-2-carboxylic acid (DHICA) or the methyl ester of DHICA (MeDHICA) at 10% *w*/*w* (panel **A**), and DHICA 1, 5, 10% *w*/*w* (panel **B**) from pristine porcine skin type A gelatin (HGel-A) in PBS at pH 7.4 with refreshing of the medium over 72 h. Reported are the mean ± SD values of three experiments and the polynomial regression that fits the data.

The kinetics of release of the indoles at physiological pH and 25 °C was then evaluated over 72 h, by refreshing the medium every hour in the first 6 h and then every 24 h. For either indoles, the release was smooth over the observation period reaching values around 30% of the incorporated indole for the 10% *w*/*w* DHICA or MeDHICa/HGel-A (Figure 2, panel A). In the case of DHICA, the release was comparable for the 5% *w*/*w* DHICA/HGel-A and up to 60% after 72 h for the 1% *w*/*w* DHICA/HGel-A (Figure 2, panel B).

**Figure 3:**
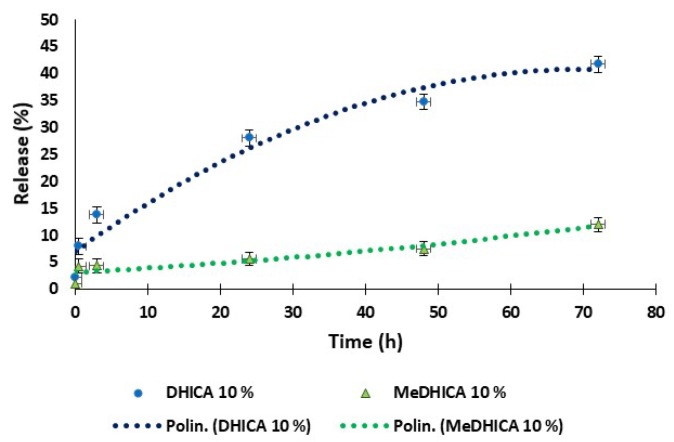
Kinetics of release of DHICA or MeDHICA at 10% *w*/*w* from HGel-A in PBS at pH 5.5 with refreshing of the medium over 72 h. Reported are the mean ± SD values of three experiments and the polynomial regression that fits the data.

The release of the indoles at 10% *w*/*w* in HGel-A was also monitored at pH 5.5, a pH value of relevance for topical delivery. A sustained release was observed for DHICA reaching 40% of the incorporated indole at 72 h, whereas this was much lower for MeDHICA (Figure 3). 

The satisfactorily high kinetics of release for DHICA also at lower pH highlights the potential use of DHICA loaded gelatin hydrogels for epidermal drug delivery in topical uses, e.g., in wound healing to ameliorate the associated inflammatory state. It is worth noting that low pHs are also found in tumor environments. Given the observed gelatin releasing ability, the proposed system may have potential as pH-dependent targeted drug delivery system in anti-cancer therapy. On the contrary, MeDHICA release is negatively affected by low pH. A possible explanation can be found in the higher solubility of the compound at pH 7.4, due to the partially ionized phenol groups. In the case of DHICA this would in part be counterbalanced by the hydrophilic character of the carboxyl group that at pH 5.5 is also ionized to a significant extent based on the pKa value of 4.25 reported for DHICA carboxyl group [41].

However, the relatively low melting temperature of gelatin hydrogels would pose severe limitations to other possible applications suggested by the biological activity of DHICA, but implying exposure to physiological temperatures. Based on this consideration, in further experiments the possibility to get a tougher material that could remain unaltered even after prolonged exposure to physiological temperature or higher was explored by two different strategies: (i) chemical cross-linking; (ii) chitosan-gelatin blends.

### 3.2. Preparation of Cross-linked Gelatins and Gelatin-Chitosan Blends

DMTMM was used as the coupling agent for gelatin cross-linking [42]. DMTMM is a zero-length coupling agent promoting the activation of carboxyl groups for subsequent amide or ester formation. Like the *N*-(3-dimethylaminopropyl)-*N*′-ethylcarbodiimide hydrochloride and *N*-hydroxysuccinimide (EDC/NHS) system for amide formation representing the standard method for zero-length cross-linking between amino and carboxyl group ligation, DMTMM is water soluble and active towards the desired reaction in a water environment. Recently, it was demonstrated that DMTMM provides better yields than the EDC/NHS system, even in the absence of pH control, that is otherwise fundamental for EDC/NHS conjugation. [42] Thus, we envisaged DMTMM as a suitable coupling agent both for pristine gelatin and for the preparation of the hydrogel composed of gelatin and chitosan, since chitosan requires acidic pH to be solubilized.

For preparation of HGel-B, the coupling agent/gelatin ratio had firstly to be optimised, starting from a 10% *w*/*v* gelatin solution in PBS. DMTMM was used in a 5, 10, 20% molar ratio with respect to total gelatin free carboxyl groups (78–80 mmol of free carboxyl groups/g of protein). The 20% DMTMM proved the only condition affording thermically stable hydrogels at 37 °C. 

In particular, HGel-B with a 1:10 or 1:5 cross-linker agent to gelatin to molar ratios were prepared and tested for DHICA/MeDHICA incorporation and release. 

HGel-C was obtained by reaction of gelatin with chitosan (≥ 75% deacetylation) in the presence of DMTMM coupling agent. 5:1 and 8:1 gelatin/chitosan *w/w* ratio were tested, using 10, 20, or 30% DMTMM molar ratio based on gelatin free carboxyl groups. Best conditions affording a stable hydrogel was the 8:1 gelatin/chitosan ratio with 10% of DMTMM. 

Both HGel-B and HGel-C proved stable at 37 °C, but also at higher temperatures up to 100 °C. 

### 3.3. Characterization of the HGel-B and HGel-C Gelatins

**Figure 4:**
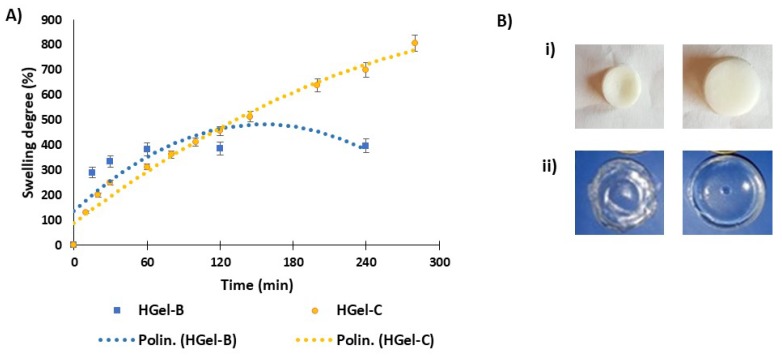
(**A**) Swelling degree over the time and (**B**) images (before and after swelling in water) of HGel-B (i) and HGel-C (ii). Reported are the mean ± SD values of three experiments and the polynomial regression that fits the data.

For both materials the swelling profile was determined in water at 25 °C. The swelling behavior expressed as swelling degree shown in Figure 4 appears rather different for the two gelatins. In the case of HGel-B the uptake of water is very rapid with respect to what observed for HGel-C. For this latter the swelling degree was found to be 800% after 6 h, whereas that of HGel-B reached 400% at 1 h. 

**Figure 5:**
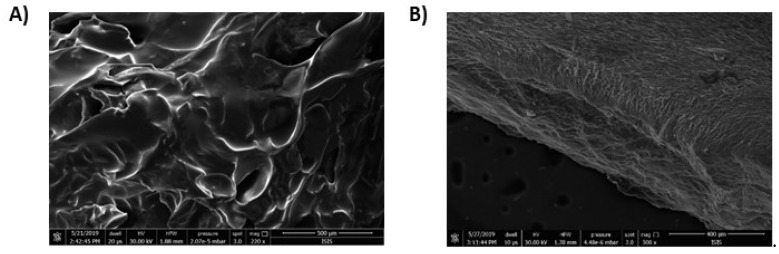
SEM images of HGel-B (**A**, scale bar 500 µM) and HGel-C (**B**, scale bar 400 µM).

The surface morphology of HGel-B and HGel-C was investigated using scanning electron microscopy (SEM, Figure 5). The two hydrogels showed quite different features. In particular, HGel-B had a rough wrinkled surface with some holes (Figure 5A), while the HGel-C exhibited a smooth membranous phase consisting of dome shaped orifices, microfibrils, and crystallite. 

**Figure 6:**
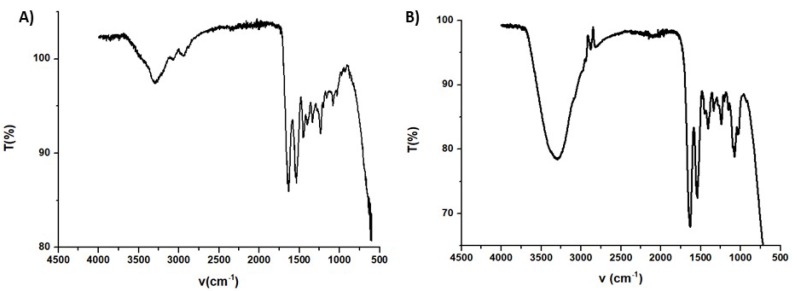
Attenuated Total Reflectance (ATR)-IR spectra of HGel-B (**A**) and HGel-C (**B**).

FT-IR spectra taken in the ATR mode for HGel-B and HGel-C are shown in Figure 6. In either cases the characteristic bands of gelatin that is the amide I and amide II stretching at 1637 and 1540 cm^-1^, respectively, and in the case of HGel-B the NH stretching band (band A) is well apparent [43]. The spectrum of HGel-C is dominated by the intense OH stretching band at around 3300 cm^-1^ in accord with the higher content of water of this sample while the well defined band at 1087 cm^−1^ (bridge C–O–C stretch) would be evidence for the presence of chitosan [44]. 

### 3.4. Loading and Release of DHICA and MeDHICA from HGel-B and HGel-C 

**Figure 7:**
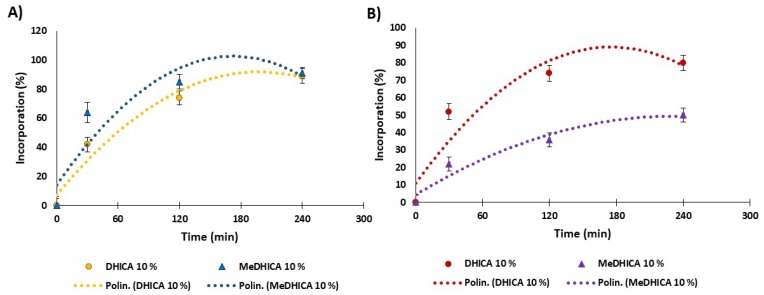
Loading of the indole compounds in the HGel-B (panel **A**) or in HGel-C (panel **B**) over time. Reported are the mean ± SD values of three experiments and the polynomial regression that fits the data.

The uptake of DHICA and MeDHICA for HGel-B is shown in Figure 7 panel A. For the HGel-B loaded at 10% *w*/*w* DHICA the uptake into the hydrogel scaffold was around 40% in the first 30 min with an increase up to 90% incorporation at equilibrium (4 h). The loading of MeDHICA 10% *w*/*w* was faster reaching 90% values in 2 h. On the other hand, the HGel-B in the presence of 5% *w*/*w* DHICA reaches 50% incorporation as the maximum value, while no appreciable loading could be detected using lower DHICA or MeDHICA to gelatin ratios (data not shown).

DHICA loading into HGel-C starting from a 10% *w*/*w* DHICA gelatin ratio appears very fast reaching a 50% incorporation in the first 30 min and up to 80% at equilibrium (4 h); on the contrary, MeDHICA incorporation was less effective, featuring only a 50% loading after 4 h (Figure 7, panel B). Again, these different behaviors may be ascribed to the free carboxy group of DHICA, that may give acid-base interactions with chitosan rich in free amino groups. These favorable interactions are not allowed with the esterified carboxy group in MeDHICA.

**Figure 8:**
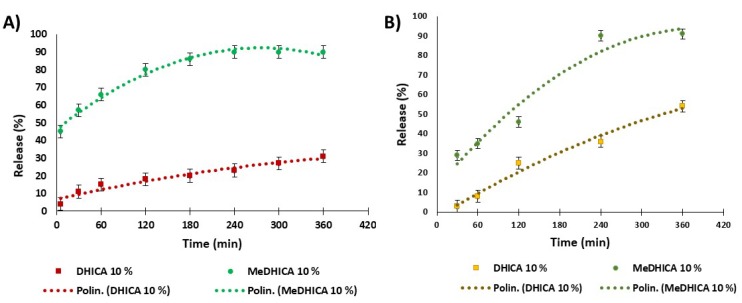
Release kinetics of the incorporated indoles from HGel-B (**A**) and HGel-C (**B**) in PBS with refreshing of the medium at 37 °C over 6 h. Reported are the mean ± SD values of three experiments and the polynomial regression that fits the data.

The release kinetics of indole derivatives was determined in PBS at 37 °C (Figure 8, panel A). In the case of 10% *w*/*w* HGel-B, the observed release of DHICA was very smooth with 15% values at 1 h up to 30% at 6 h with medium refreshing every 1 h, and no significant changes for longer times up to 24 h. The release of DHICA is even lower (23% at 6 h) with 5% *w*/*w* HGel-B loaded with the indole, (data not shown). MeDHICA in 10% *w*/*w* HGel-B is released rapidly (57% after 1 h), up to 90% after 4 h with repeated medium refreshing. Much slower release kinetics was observed for DHICA in the case of HGel-C with an initial value of 8% after 1 h which increases up to 54% after 6 h with medium refreshing. On the other hand, MeDHICA is released to 35% after 1 h with a steep increase up to 90% in the first 4 h after repeated medium refreshing (Figure 8, panel B).

In summary (Table 1), loading and release values seem to be dictated mainly by the interactions between the gel and the indoles carboxy groups, and to a lesser extent by the acidic phenolic functionalities. The lower incorporation of DHICA in HGel-A is likely due to the unfavorable interactions of the carboxylate group of DHICA with the carboxylate groups of the acidic aminoacids of gelatin. Such an effect would be decreased with HGel-B and HGel-C, where gelatin carboxy groups are capped by the cross-linking reaction and by the increase of basic amino groups from chitosan, respectively. On the contrary, MeDHICA, missing the ionizable and strongly hydrophilic carboxy group does not experience unfavorable interactions with pristine gelatin (HGel-A) and HGel-B as indicated by the high loading extent, but has a lower affinity for the hydrophilic HGel-C, as indicated also by the higher extent of release with respect to DHICA.

### 3.5. Assessment of the Stability of DHICA in the HGel-B and -C Gelatins

One of the advantages that should be offered by incorporation of melanin related indoles into a biopolymer like gelatin is the increase of the stability to aerial oxidation in aqueous neutral media of physiological relevance. This issue is more critical in the case of DHICA with respect to MeDHICA given its higher proness to oxidation as shown in our previous studies [36].

**Figure 9:**
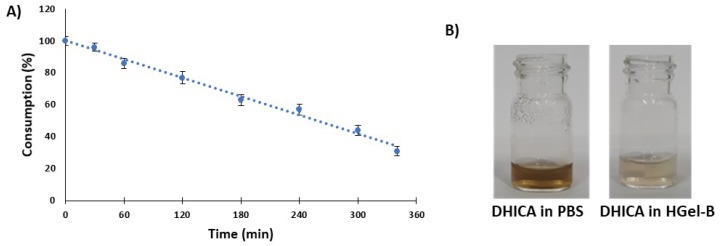
Panel **A**: Decay of free DHICA in the PBS solution monitored by HPLC analysis. Panel **B**: Appearance of the free DHICA solution in PBS (left) vs. the 10% *w*/*w* DHICA/HGel-B containing medium (right) over 6 h. Reported are the mean ± SD values of three experiments.

Therefore, in subsequent experiments the kinetics of decay of free DHICA in PBS at 37 °C was evaluated by HPLC analysis over the time period used for monitoring the release from cross-linked gelatins. HGel-B loaded with DHICA at 10% was immersed in PBS at 37 °C and the release was again monitored over 6 h without medium refreshing leading to a release of 10% of the incorporated compound. The decay of free DHICA in the PBS solution at the same concentration estimated based on the incorporation shown in Figure 7, panel A, was monitored by HPLC analysis. Figure 9 (panel A) shows that under these latter conditions DHICA was consumed to 70% over 6 h being oxidized to dark melanin, whereas the indole entrapped into the gelatin and hence released slowly into solution was preserved from oxidation to a remarkable extent (Figure 9, panel B).

### 3.6. Evaluation of the Antioxidant Properties

**Figure 10:**
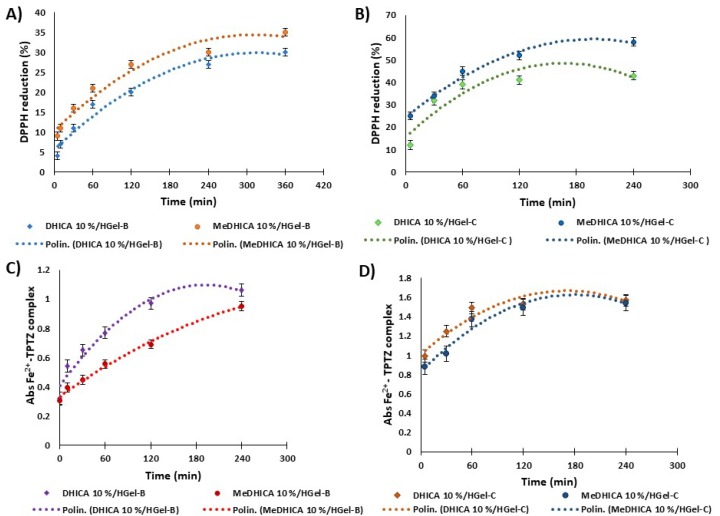
2,2-diphenyl-1-picrilhydrazyl (DPPH) reduction properties of DHICA or MeDHICA 10% HGel-B (panel **A**) and 10% HGel-C (panel **B**) over time; increase of the absorbance at 593 nm due to the Fe^2+^ TPTZ complex induced by DHICA or MeDHICA 10% HGel-B (panel **C**) and 10% HGel-C (panel **D**). Reported are the mean ± SD values of three experiments and the polynomial regression that fits the data.

Considering the remarkable antioxidant activity of the compounds under investigation, the antioxidant properties of the DHICA/MeDHICA released in PBS at 37 °C from the 10% HGel-B and HGel-C were also evaluated by the DPPH and FRAP assays. Briefly, aliquots of the medium were withdrawn over time and the DPPH decay after 10 min was evaluated spectrophotometrically (Figure 10, panels A and B). A similar procedure was followed to evaluate the ferric reducing antioxidant power of the medium containing the 10% HGel-B and HGel-C (Figure 10, panels C and D). As expected, the reducing potency increased over time as a result of the progressive release of the indoles from the hydrogels further confirming the observed stability of the indole compounds incorporated into the hydrogels.

The antioxidant potency of HGel-C appeared higher than that of HGel-B in either assays. This result can be interpreted considering the higher release of the indoles in terms of rate and extent from HGel-C with respect to HGel-B. Previous studies on the antioxidant effect of the two indoles have shown an around 2.5-fold higher activity for DHICA with respect to MeDHICA in the DPPH assay [36]. This result allows to rationalize the effect on DPPH consumption observed for DHICA and MeDHICA incorporated into HGel-B (28 and 32%, respectively), in the light of the concentration of the indoles actually released in incubation medium after 6 h, that is 9 μM and 19 μM for DHICA and MeDHICA, respectively. This holds also for HGel-C with a 40 and 55% DPPH consumption for DHICA and MeDHICA, respectively, considering that DHICA and MeDHICA are present in solution at concentrations of 13 μM and 31 μM, respectively. Given the temperature conditions of these experiments HGel-A could not be tested. 

**Figure 11:**
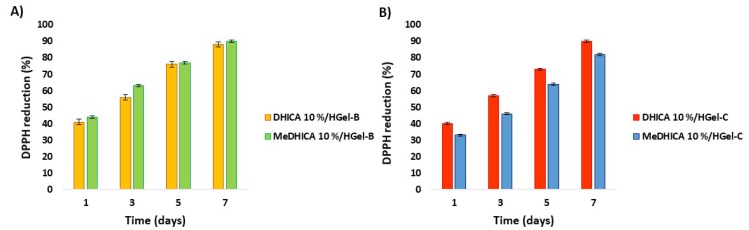
Reduction of DPPH by HGel-B (**A**) or HGel-C (**B**) loaded with DHICA or MeDHICA at 10%. Reported are the mean ± SD values of three experiments.

The antioxidant potency of DHICA or MeDHICA incorporated into the gelatin hydrogels persisted even after prolonged storage of the material. This issue was demonstrated by DPPH assay applied to indole loaded HGel-B and HGel-C that have been exposed to air over one week. The reducing ability of the materials immersed in the 200 µM DPPH solution at a 0.04 *w*/*v* ratio at 25 °C increased over time reaching an almost complete consumption of the reagent after 7 days (Figure 11). The extent of incorporation affected the antioxidant effects. In agreement with the higher incorporation of DHICA in HGel-C the antioxidant activity of the loaded gelatin appeared slightly higher than that of MeDHICA/HGel-C, even if the higher solubility of MeDHICA in the assay medium (methanol) would have favored it compared to DHICA. Control experiments proved the stability of the DPPH reagent over the period of the assay. Moreover, the DPPH consumption profile of the loaded gelatins that had not been subjected to storage proved closely similar to that observed for the gelatins exposed to air over one week. For instance, in the case of HGel-B loaded with DHICA, a DPPH consumption of 86% is observed after 7 days. A DPPH consumption of 90% after seven days was obtained also for HGel-A that could be tested under these experimental conditions.

## 4. Conclusions

Gelatin based hydrogels are currently the focus of much interest in different fields of biomedical relevance. Even the raw material from different sources including fish skin has been described to have a potential for cell therapies for cutaneous wound, [24] whereas crosslinked gelatin hydrogels or gelatin blends hydrogel have been developed for three-dimensional (3D) cell culture platform [45]. The possibility to use gelatin based hydrogels as carrier for the controlled release of biologically active unstable compounds further adds to the opportunities offered by this system. In this frame the present work explored the potential of gelatin from porcine skin and cross-linked gelatin (HGel-B) or chitosan/gelatin blends (HGel-C) to incorporate DHICA, a melanin-related metabolite endowed with marked antioxidant and cell differentiation stimulation activity and its methyl ester, a more stable compound with a remarkable antioxidant profile. Both indole compounds were effectively incorporated into the pristine gelatin hydrogels and were smoothly released in PBS at pH 7.4, whereas only DHICA was released to a significant extent at pH 5.5, an observation of relevance for the implementation of devices for topical applications. Different incorporation and release profiles where exhibited by the two indoles for HGel-B and HGel-C. The higher affinity of DHICA for HGel-C likely reflects the higher hydrophilic character of the indole and of the cross-linked gelatins itself containing substantial amounts of chitosan as evidenced by FT-IR analysis. More interestingly, gelatin-based hydrogels offer protection to DHICA against air oxidation. Indeed, the cross-linked gelatins incorporating the indoles exhibited a marked antioxidant activity that persisted overtime and was not lost even after prolonged storage of the material. All together these results highlight gelatins as easily accessible biocompatible materials that could warrant a sustained release of labile bioactives under physiologically relevant conditions. The variability of the loading extent and kinetics of release of the three gelatin types investigated would suggest that proper modification of the raw material can allow tuning of the time scale of delivery for different therapeutical applications. 

## Figures and Tables

**Table 1:** Overview of the percentage values of loading and release of the indole compounds from the gelatin hydrogels investigated.

	Loading (%, at Equilibrium)			Release (%)		
	HGel-A	HGel-B	HGel-C	^a^ HGel-A	^b^ HGel-B	^b^ HGel-C
DHICA (10% *w*/*w*)	62 ± 1.7	90 ± 2.2	80 ± 2.2	30 ± 0.57	30 ± 1.6	54 ± 1.6
MeDHICA (10% *w*/*w*)	80 ± 1.3	88 ± 1.4	50 ± 2.1	28 ± 0.8	90 ± 1.4	90 ± 1.2

^a^ Release evaluated in PBS 1× pH 7.4 at room temperature; ^b^ Release evaluated in PBS 1× pH 7.4 at 37 °C.
